# PerPlot & PerScan: tools for analysis of DNA curvature-related periodicity in genomic nucleotide sequences

**DOI:** 10.1186/2042-5783-1-13

**Published:** 2011-11-28

**Authors:** Jan Mrázek, Tejas Chaudhari, Aryabrata Basu

**Affiliations:** 1 Department of Microbiology and Institute of Bioinformatics, University of Georgia, Athens, GA 30602-2605, USA; 2 Department of Computer Science, University of Georgia, Athens, GA 30602-7404, USA

**Keywords:** Software, Genome, Chromosome, DNA structure, Nucleoid, Chromatin, A-tracts, Periodic spacing, Gene expression

## Abstract

**Background:**

Periodic spacing of short adenine or thymine runs phased with DNA helical period of ~10.5 bp is associated with intrinsic DNA curvature and deformability, which play important roles in DNA-protein interactions and in the organization of chromosomes in both eukaryotes and prokaryotes. Local differences in DNA sequence periodicity have been linked to differences in gene expression in some organisms. Despite the significance of these periodic patterns, there are virtually no publicly accessible tools for their analysis.

**Results:**

We present novel tools suitable for assessments of DNA curvature-related sequence periodicity in nucleotide sequences at the genome scale. Utility of the present software is demonstrated on a comparison of sequence periodicities in the genomes of *Haemophilus influenzae*, *Methanocaldococcus jannaschii*, *Saccharomyces cerevisiae*, and *Arabidopsis thaliana*. The software can be accessed through a web interface and the programs are also available for download.

**Conclusions:**

The present software is suitable for comparing DNA curvature-related sequence periodicity among different genomes as well as for analysis of intrachromosomal heterogeneity of the sequence periodicity. It provides a quick and convenient way to detect anomalous regions of chromosomes that could have unusual structural and functional properties and/or distinct evolutionary history.

## Background

Most naturally occurring DNA sequences feature two strong periodic patterns. The first relates to a 3 bp period resulting from amino acid and codon usage biases in protein coding genes. The second arises from periodic spacing of A-tracts (short runs of A or T) phased with the DNA helical period of ~10.5 bp. The periodically spaced A-tracts are a primary indicator of intrinsically bent DNA and the main component of nucleosome positioning signals in eukaryotes [1-3]. Similar periodic patterns are present in prokaryotes, where they could contribute to DNA packaging in the nucleoid [4,5], promote the appropriate mode of supercoiling [6,7], and/or facilitate the initiation and termination of transcription [8,9]. There are significant differences in the character and intensity of these periodic patterns among different genomes as well as among different segments of the same genome [4,6,7,10]. In some species, the intragenomic heterogeneity of the sequence periodicity has been linked to local variance in gene expression and chromatin structure [4,11,12].

Despite the biological significance of DNA curvature-related sequence periodicity, there are virtually no online tools available for analysis of these periodic signals at the genome scale. We present interfaces to Periodicity Plot (PerPlot) and Periodicity Scan (PerScan) tools, based on the methodology that was initially developed for comparative analyses of prokaryotic genomes [4]. PerPlot detects predominant periodicities in a nucleotide sequence, whereas PerScan can be used to analyze intrachromosomal heterogeneity of the periodic signal. Postprocessing options include a capability to extract genes and other annotated sequence features located in strongly periodic or non-periodic sections of the chromosome. Although initially designed for analysis of prokaryotic genomes, the software can also be applied to complete eukaryotic chromosomes.

## Implementation

### PerPlot

The program starts by counting the number *N*(*s*) of times a pair of A-tracts occur in the analyzed sequence at a mutual distance *s*. This initial step is similar to the approach previously used by Herzel and coworkers [6,7]. Users can choose from ten alternative definitions of A-tracts, starting with a single A or T, extending to short uninterrupted runs of A or T of lengths 2-5 bp, and short oligonucleotides composed of A's followed by T's (that is, containing only the dinucleotides AA, AT, and TT) [3,5]. Restricting the periodicity analysis to such A-tracts is justified because these sequences have a dominant effect on DNA curvature and exhibit strong periodic spacing in many different genomes [3-5,7,13-16]. The function *N*(*s*) is subsequently normalized relative to expected counts and further processed to reduce artifacts unrelated to DNA curvature. The 3-bp periodic signal arising from biased codon usage in genes is removed with a 3-bp sliding window average and a slope in the plot that can arise from heterogeneity of G+C content is eliminated by subtracting a parabolic regression from the observed values.

A section of the modified *N*(*s*) function in the range determined by user-defined parameters *s*_min _and *s*_max _is converted to a power spectrum with the Fourier transform. The default values for *s*_min _and *s*_max _are set to 30 and 100 bp, respectively. Setting *s*_min _to 30 bp eliminates most of the periodic signal that can arise from amphipathic α-helices in the encoded proteins while the selection of 100 bp for *s*_max _follows from the observation that the periodic signal in many genomes does not extend beyond ~150 bp [4,5,7,15]. The power spectrum is subsequently scaled to average 1 over the range of periods between 5 and 20 bp. This normalization allows comparing the heights of the peaks for sequences of varying lengths and oligonucleotide compositions. Assuming that most of the periods in the 5-20 bp range do not carry a significant periodic signal, the mean value of the power spectrum over a range of periods can be used as a measure of random noise in the spectrum, which is an appropriate normalization factor. We refer to such normalized power spectrum as "periodicity plot" and we formally designate it as Q*(*P*) - a normalized measure of the intensity of the periodic signal as a function of the period *P*.

**Figure 1:**
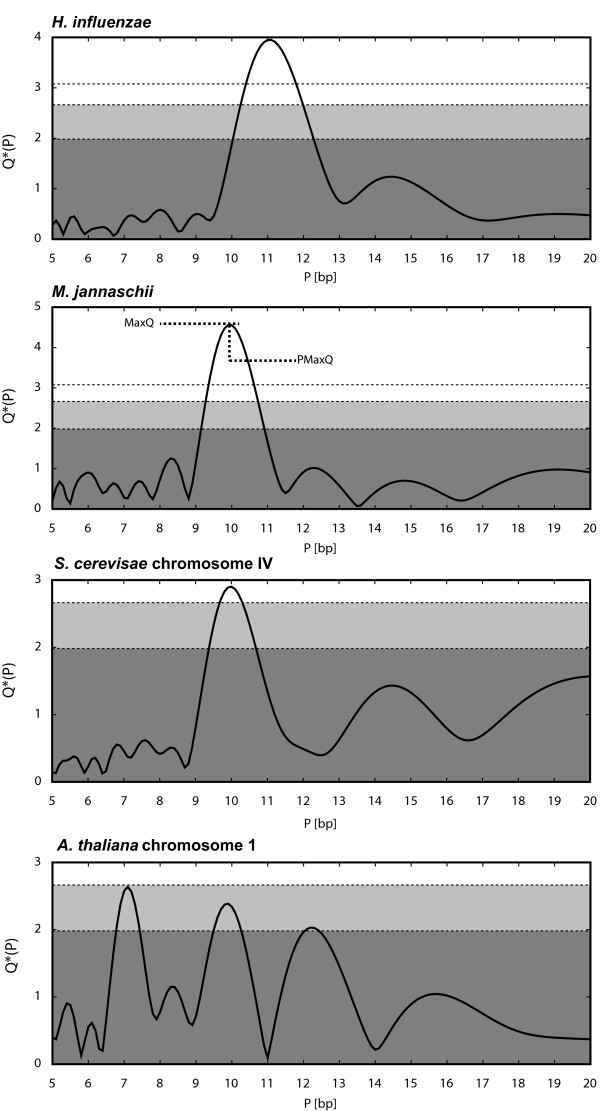
**Periodicity plots for the four analyzed genomes**. The ordinate displays the normalized intensity Q*(*P*) of the periodic signal in the spacing of AA and TT dinucleotides for the period shown by the abscissa. The parameters *s*_min _and *s*_max _were set at 30 and 100 bp, respectively. See text for details. The horizontal lines and shading refer to statistical significance of the peaks in the plot. The dark shaded area corresponds to values below the 50^th ^percentile of the dominant periodic signal in random sequences. The light shaded area refers to values between the 50^th ^and 95^th ^percentiles. Peaks rising above the shaded area can be considered statistically significant. An additional line without shading refers to the 99^th ^percentile. The definition of the MaxQ and PMaxQ indices is demonstrated in the periodicity plot for *M. jannaschii*.

The program also outputs two indices that characterize the periodicity of the analyzed sequence: the height of the dominant peak (MaxQ) and the period corresponding to the dominant peak (PMaxQ) (Figure 1). These indices are suitable for comparisons among large numbers of different genomes where comparing the whole plots would be impractical [4]. The PerPlot output includes the relevant plots in PostScript and/or PDF formats, the same data tabulated in a tab-delimited text file, and the MaxQ and PMaxQ indices.

### Assessments of statistical significance

Small peaks in the periodicity plot can arise from stochastic noise. To help users assess the statistical significance of the periodic signals detected by PerPlot we performed extensive simulations with random sequences and used these results to indicate significance of the peaks in the output. We used a dataset of 1025 complete prokaryotic chromosomes [4] and generated 20 random sequences matching the length and nucleotide composition of each chromosome. This resulted in a collection of 20,500 random sequences. The random sequences were generated using the homogeneous Bernoulli model implemented in the Genome Randomizer software [17](http://www.cmbl.uga.edu/software.html). The MaxQ index was determined for each random sequence using 50 different combinations of user-defined parameters: ten different A-tract definitions and five different values of the difference *s*_max_-*s*_min_. Because the random sequences were generated as strings of independently drawn letters the distribution of MaxQ values in the random sequences does not depend on specific values of *s*_min _and *s*_max _but only on the difference *s*_max_-*s*_min_. For each combination of the A-tract definition and the *s*_max_-*s*_min _difference, we determined the 50^th^, 95^th^, and 99^th ^percentiles of the MaxQ values among the 20,500 simulations (Table 1). These percentiles are shown in the PerPlot output and can serve as guidance in assessing the significance of peaks in the periodicity plot (Figure 1). Linear interpolation is used to determine the percentiles for *s*_max_-*s*_min _values different from those for which the percentiles were determined directly. These estimates do not take into account the differences in sequence lengths because the MaxQ distributions in random sequences do not vary over the range of lengths characteristic of microbial chromosomes (data not shown). We also noted that the G+C content of the random sequences does not affect the MaxQ values, although there is a relationship between A-tract periodicity and G+C content in "real" genomes [4].

**Table 1:** MaxQ index percentiles in random sequences^a^.

Method^b^	MaxQ percentiles for five different spacing ranges^c^				
	
	40 bp	70 bp	100 bp	150 bp	200 bp
AT	3.07, 2.57, 1.80^d^	3.15, 2.71, 1.99	3.18, 2.79, 2.10	3.26, 2.89, 2.23	3.32, 2.96, 2.31
A2T2	2.98, 2.52, 1.80	3.08, 2.66, 1.98	3.17, 2.77, 2.09	3.26, 2.90, 2.23	3.36, 2.98, 2.32
A3T3	2.89, 2.50, 1.80	3.05, 2.65, 1.99	3.17, 2.77, 2.11	3.28, 2.90, 2.24	3.35, 2.99, 2.33
A4T4	2.90, 2.45, 1.79	3.03, 2.64, 1.99	3.15, 2.76, 2.11	3.27, 2.91, 2.24	3.39, 3.01, 2.34
A5T5	2.81, 2.42, 1.77	2.96, 2.60, 1.96	3.11, 2.73, 2.09	3.21, 2.88, 2.23	3.33, 2.98, 2.33
AT2	2.97, 2.50, 1.79	3.08, 2.66, 1.98	3.16, 2.76, 2.10	3.24, 2.88, 2.23	3.32, 2.96, 2.31
AT3	2.94, 2.48, 1.80	3.07, 2.66, 1.98	3.15, 2.77, 2.11	3.28, 2.90, 2.24	3.37, 3.00, 2.33
AT4	2.88, 2.47, 1.79	3.05, 2.65, 1.99	3.17, 2.77, 2.12	3.27, 2.91, 2.25	3.39, 3.01, 2.35
AT5	2.90, 2.45, 1.79	3.01, 2.62, 1.99	3.14, 2.76, 2.11	3.27, 2.92, 2.26	3.40, 3.02, 2.35
AT6	2.78, 2.40, 1.75	2.96, 2.59, 1.94	3.08, 2.73, 2.07	3.24, 2.88, 2.21	3.35, 2.98, 2.31

^a ^MaxQ measures the highest periodic signal intensity detected over the range or periods 5-20 bp. The table shows the 99^th^, 95^th^, and 50^th ^percentile, for each combination of parameters. See text for details.

^b ^Definition of A-tracts: "AT", single nucleotides A or T; "A2T2" dinucleotides AA or TT; "AT2", dinucleotides AA, AT, or TT; "A3T3" trinucleotides AAA or TTT; "AT3", trinucleotides AAA, AAT, ATT, or TTT; etc.

^c ^The spacing range refers to the difference between parameters *s*_max_-*s*_min_. The simulations were performed for spacing range values 40, 70, 100, 150, and 200 bp. See text for details.

^d ^The 99^th^, 95^th^, and 50^th ^percentiles, respectively, of the MaxQ values in 20,500 random sequences are shown.

### PerScan

**Figure 2:**
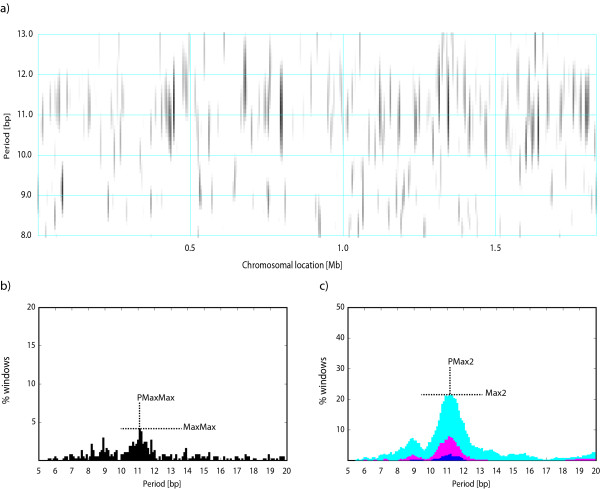
**Periodicity scan of the *H. influenzae *chromosome**. a) The main periodicity scan plot. The level of grey signifies the intensity of the periodic signal for the chromosomal location shown on the horizontal axis and the period shown on the vertical axis. The periodicity was evaluated in a 10 kb window was shifted by 5 kb at a time. The white areas correspond to the relative signal intensity Q*(*P*)≤1.8 whereas black shading indicates signal intensity Q*(*P*)≥4.0. The level of gray continuously changes from white to black between the values 1.8 and 4.0. b) The fraction of windows with the maximum signal at the period indicated by the abscissa plus or minus 0.2 bp, regardless of the height of the maximum. c) The fraction of windows with the signal intensity for the given period Q*(*P*)≥2.0 (cyan), ≥2.5 (magenta), ≥3.0 (blue), ≥4.0 (green), and ≥6.0 (red). See text for details. The definitions of indices MaxMax, PMaxMax, Max2, and PMax2 are demonstrated in panels b and c. The indices Max3 and PMax3 are analogous to Max2 and PMax2 but derived from the blue section of the plot.

The PerPlot technique described above can detect a presence of a periodic signal in the analyzed DNA sequence but it does not provide any information about the distribution of the signal along the sequence. For example, the question whether the periodicity is uniformly distributed along the sequence or concentrated in a few chromosomal regions with strong sequence periodicity can be important for the interpretation of the observed periodic patterns [4]. To investigate the intrachromosomal heterogeneity of the periodic patterns we designed PerScan, which applies the PerPlot technique in a sliding window. The main output is a heat map where the level of gray in the plot area indicates the intensity of the periodic signal with the period shown on the vertical axis and the window location determined by the horizontal axis. We refer to this plot as "periodicity scan". Additional plots show the percentage of sliding window locations that exhibit a periodic signal of a specified minimum intensity, which is a useful indicator of the persistency of the periodic signal throughout the analyzed DNA sequence (Figure 2). Three pairs of indices MaxMax and PMaxMax, Max2 and PMax2, and Max3 and PMax3 are derived from these plots, which measure the persistency of the dominant periodic signal (Figure 2). The output includes the plots in PostScript and/or PDF formats, the same data in a tabulated format (a large tab-delimited text file), and the periodicity indices. A detailed description of the methodology utilized in PerPlot and PerScan is available online http://www.cmbl.uga.edu/software/Perplot_HTML/Perplothtml.html, http://www.cmbl.uga.edu/software/PerScan_HTML/perscanhtml.html and in ref. [4]. Both PerPlot and PerScan include an option to mask out the protein coding sequences (CDS features in GenBank files) or noncoding sequences (all sequences not labeled CDS in the GenBank-formatted input files).

### Postprocessing

If the analyzed sequence is provided in the GenBank format with annotation, the users can further process the PerScan output to extract annotated features that overlap with windows exhibiting unusually high or low sequence periodicity. All annotated features that overlap with windows that have maximum periodicity over a given range of periods higher or lower (the user's choice) than a user-defined cutoff are listed in the output. Two output files are generated: one contains a filtered features table from the original GenBank file in the original GenBank format. The second output is a user-friendly tab-delimited file with less information. The users can perform multiple rounds of postprocessing with the same PerScan output.

### Implementation

The PerPlot and PerScan tools are hosted on a multiprocessor workstation utilizing the Apache server and Redhat Enterprise Linux. CGI interfaces along with programs in C and scripts in Python constitute the software environment. A database of complete prokaryotic genomes is stored locally and shared with other web services provided by the same server. The database is periodically synchronized with the list of complete genomes at the National Center for Biotechnology Information (ftp://ftp.ncbi.nih.gov/genomes/). Users can select the sequence files from the local database or upload their own input files. The uploaded sequences must be in GenBank or FASTA format, and contain only one sequence entry per file. All output files are stored in a unique directory created on the server for that session and kept for at least 30 days. The output files are not password-protected but the uploaded sequences are stored separately in a secure area. Prokaryotic genomes are typically processed in a few seconds using the default parameters. However, uploading large files for analysis can take several minutes depending on the network speed.

## Results and Discussion

We demonstrate the use of the PerPlot and PerScan tools by comparing the periodic patterns in the genomes of a bacterium *H. influenzae*, an archaeon *M. jannaschii*, the largest chromosome of the yeast *S. cerevisiae *(chromosome IV), and the *A. thaliana *chromosome 1. The first three sequences were selected because they are similar in length (1.83 Mb, 1.66 Mb, and 1.53 Mb, respectively) and also because they represent the first completely sequenced genomes in each domain of life [18-20]. In addition, the protein-coding DNA fraction is not dramatically different among the three genomes-about 87% in *H. influenzae*, 88% in *M. jannaschii*, and 73% in *S. cerevisiae*. The *A. thaliana *chromosome 1 was included as a representative of higher eukaryotes [21]. All data presented here refer to spacings between pairs of AA and TT dinucleotides (the "A2T2" method) and the spacing range 30-100 bp (the default *s*_min _and *s*_max _parameters).

Figure 1 shows the periodicity plots for the four analyzed chromosomes. The *H. influenzae *and *M. jannaschii *chromosomes exhibit strong periodic signals at periods about 11 and 10 bp, respectively. The difference in the predominant 10 or 11 bp periodicity is consistent with a previously observed distinction between bacteria and a subset of archaea [4,6,7]. The yeast chromosome shows a weaker but still significant peak at the period 10 bp. It is interesting to note that although sequence periodicity is often associated with nucleosome positioning in eukaryotes [13], the periodic patterns as assessed by PerPlot and similar methods are generally stronger in prokaryotes than in most eukaryotic genomes (ref. [7] and data not shown). The periodicity plot for the *A. thaliana *chromosome 1 does not exhibit any peaks exceeding the 95^th ^percentile significance threshold (the shaded area). Moreover, the highest peak corresponds to a 7 bp period, which is unrelated to DNA curvature. This peak is caused by tandem heptanucleotide repeats and disappears when the tandem repeats are masked out or when the analysis is restricted to protein-coding regions, leaving a dominant peak at the period ~10 bp (data not shown).

**Figure 3:**
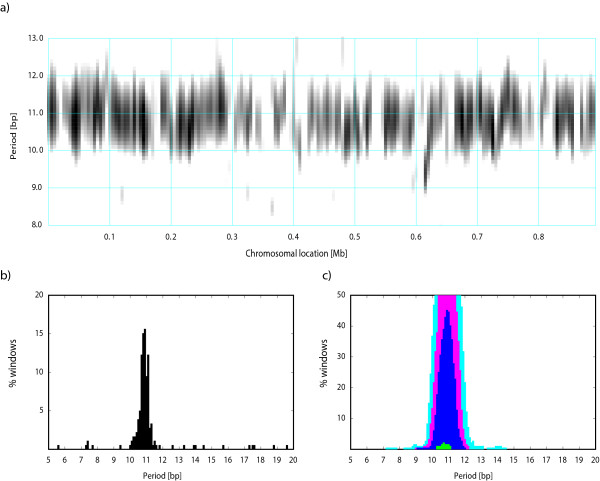
**Periodicity scan of the *M. hyopneumoniae *chromosome**. See legend to Figure 2.

We subsequently used the PerScan tool to investigate intrachromosomal heterogeneity of the periodic signals. We performed the analysis with a sliding window of 10 kb which was moved at steps of 5 kb at a time. Figure 2 shows the periodicity scan for *H. influenzae*. Consistent with the periodicity plot in Figure 1, the 11 bp periodicity dominates the periodic regions. However, the periodicity scan shows that the periodic signal is mostly concentrated in a few short regions while most of the genome shows little sequence periodicity. Less than 10% of the chromosome exhibits a periodic signal with strength ≥2.5, which is close to the 95^th ^percentile in random sequences (Figure 2c). Such heterogeneity of the periodic signal is typical of most genomes, although some genomes exhibit very persistent periodic signal throughout the chromosome (see Figure 3 for *Mycoplasma hyopneumoniae *232) [4]. We used the postprocessing of the PerScan results to identify the *H. influenzae *genes overlapping with segments that had the periodic signal intensity ≥3.5 for periods 10.6-11.6 bp (the dominant period 11.1 bp plus or minus 0.5 bp). There are three such segments located near positions 450 kb, 800 kb, and 1350 kb. The genes located in these chromosomal segments include several metabolic enzymes, DNA polymerase and gyrase subunits, and hypothetical proteins (Table 2).

**Table 2:** *H.influenzae *genes located in regions with a strong sequence periodicity.

Locus tag	Start	End	Strand	Product
HI0417	439370	440050	+	thiamine-phosphate pyrophosphorylase ThiE
HI0418	439995	441338	+	transport protein
HI0419	441507	442889	+	protease
HI0420	443031	443330	+	hypothetical protein
HI0422	444029	445348	-	ATP-dependent RNA helicase SrmB
HI0423	445394	446116	+	hypothetical protein
HI0424	446149	447204	-	rRNA methylase
HI0425	447351	448718	+	phosphatidylserine synthase PssA
HI0426	448763	449488	-	fatty acid metabolism regulator FadR
HI0427	449613	451157	+	sodium/proton antiporter NhaB
HI0736	789998	791524	-	sodium-dependent transporter
HI0737	791772	792569	+	acetohydroxy acid synthase II
HI0738	792641	794479	+	dihydroxy-acid dehydratase IlvD
HI0738.1	794559	796100	+	threonine dehydratase IlvA
HI0739	796139	799618	-	DNA polymerase III subunit alpha DnaE
HI0740	799857	801509	+	Phosphomannomutase YhxB
HI1262	1339751	1340431	-	SanA
HI1263	1340589	1341665	+	homoserine O-acetyltransferase MetX
HI1264	1341719	1344361	-	DNA gyrase subunit A GyrA
HI1265	1344944	1346707	-	hypothetical protein
HI1266	1346844	1347230	-	hypothetical protein
HI1268	1347455	1347634	+	hypothetical protein
HI1269	1347628	1347744	+	hypothetical protein
HI1272	1348468	1349259	+	ABC transporter ATP-binding protein
HI1273	1349256	1350062	+	hypothetical protein

**Figure 4:**
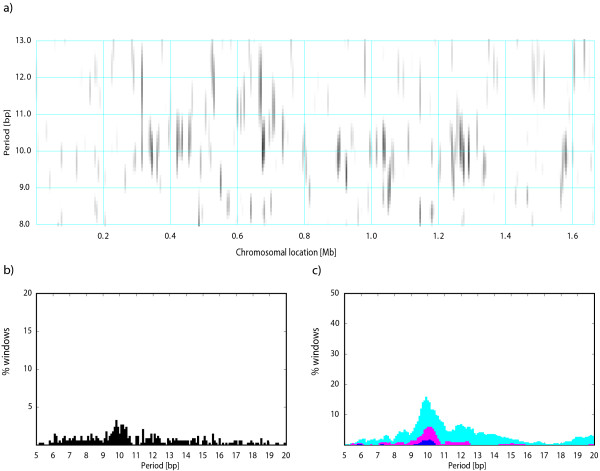
**Periodicity scan of the *M. jannaschii *chromosome**. See legend to Figure 2.

*M. jannaschii *shows a similarly heterogeneous periodic signal but with most periodic segments exhibiting the periodicity ~10 bp, which is characteristic of some archaeal genomes [4,6,7] (Figure 4). Several chromosomal regions also show periodicity between 11 and 12 bp. Note that the periodicities at 12 bp as well as other multiples of three can arise from amino acid repeats in proteins and may not be related to DNA curvature. Presence of segments with the 10-bp and 11-bp periodicities could indicate lateral gene transfer between bacteria, which typically have an 11-bp dominant period, and archaea, which often exhibit a 10-bp periodicity. Such dual periodicity was observed in the genome of the bacterium *Thermotoga maritima*, which contains many genes of apparent archaeal origin [10]. The region around position 710 kb in the *M. jannaschii *chromosome shows a dominant periodicity of ~11 bp (Figure 4), which might indicate a possible bacterial origin of some genes in this region. Genes located in this chromosomal segment are listed in Table 3 along with the top three BLAST hits outside the order Methanococcales. The top BLAST hits are almost exclusively to archaeal genes, which is not indicative of lateral transfer from bacteria, suggesting that the ~11 bp periodicity in this case does not relate to a bacterial origin of this DNA segment. Herzel et al. [6,15] proposed a relationship of the 10 bp and 11 bp periodicity with a positive and negative supercoiling, respectively; in this regard the 11-bp-periodic region could indicate a negatively supercoiled segment in an otherwise predominantly positively supercoiled chromosome.

**Table 3:** *M. jannaschii *genes located in the region with 11 bp periodicity.

Locus tag	Start	End	Strand	Product	Species with top three BLAST hits^a^
MJ0782	703765	705786	-	transcription initiation factor IIB	*Methanobacterium *sp. *Methanobrevibacter ruminantium* *Methanothermobacter thermoautotrophicus*

MJ0782.1	705793	706038	-	H/ACA RNA-protein complex component Gar1	*Methanopyrus kandleri* *Halorubrum lacusprofundi* *Methanothermobacter thermoautotrophicus*

MJ0783	706179	706739	+	hypothetical protein	*Acidianus hospitalis* Thermococcus sibiricus *Clostridium stricklandii

MJ0784	707015	708091	+	H(2)-dependent methylenetetrahydro-methanopterin dehydrogenase	*Methanothermus fervidus* *Methanoplanus petrolearius* *Methanothermobacter thermoautotrophicus*

MJ0785	708313	709440	+	biotin synthase	*Methanothermus fervidus* *Methanothermobacter thermoautotrophicus* *Methanothermobacter marburgensis*

MJ0785.1	709430	709999	+	hypothetical protein	*^+^Karlodinium micrum *chloroplast *Saccharophagus degradans

MJ0786	710062	710622	+	hypothetical protein	*Methanohalobium evestigatum* *Methanohalophilus mahii* *Methanococcoides burtonii*

MJ0787	710772	712286	+	hypothetical protein	*Methanothermus fervidus* *Methanobrevibacter smithii* *Methanothermobacter marburgensis*

MJ0788	712302	712541	+	hypothetical protein	*Methanosarcina mazei* *Methanoplanus petrolearius* *^+^Neosartorya fischeri*

MJ0789	712624	712974	+	hypothetical protein	*Methanohalophilus mahii* *Methanosarcina mazei* *Methanosarcina barkeri*

MJ0790	713009	713698	+	NADH dehydrogenase subunit 1	*Methanothermus fervidus* *Methanothermobacter thermoautotrophicus* *Methanothermobacter marburgensis*

MJ0791	713720	715174	-	argininosuccinate lyase	*Methanothermus fervidus* *Methanothermobacter marburgensis* *Methanothermobacter thermoautotrophicus*

^a ^Excluding the order Methanococcales. Only one hit per species is reported (excluding hits to multiple strains). Hits to eubacterial proteins are labeled by an asterisk and hits to eukaryotes or organelles by a "+". The blastp program implementation at the NCBI web site http://blast.ncbi.nlm.nih.gov with default parameters was used to find the top hits. Fewer than three hits are shown when less than three significant hits were detected.

**Figure 5:**
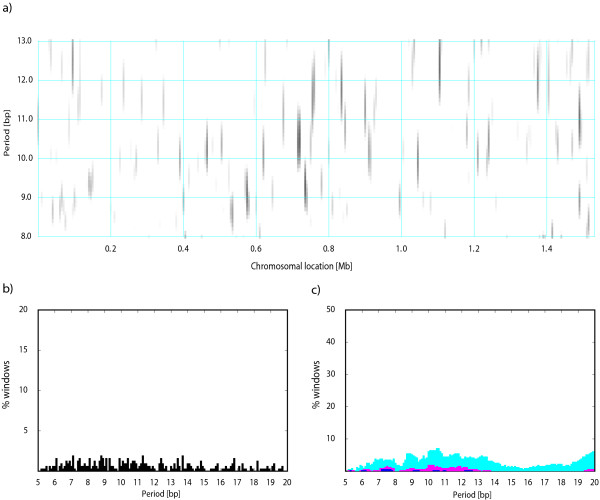
**Periodicity scan of the *S. cerevisiae *chromosome IV**. See legend to Figure 2.

Most of the *S. cerevisiae *chromosome IV is devoid of a detectable periodic signal (Figure 5). The strongest periodicity is detected in regions 715-740 kb (including genes YDR129C-YDR141C) and 1485-1495 kb (genes YDR522C-YDR528W) with predominant periodicities of ~10.5 and ~11 bp, respectively. Genes in these regions encode mostly proteins involved in cytoskeleton, transcription, signal transduction, and sporulation, in addition to several hypothetical proteins (data not shown).

**Figure 6:**
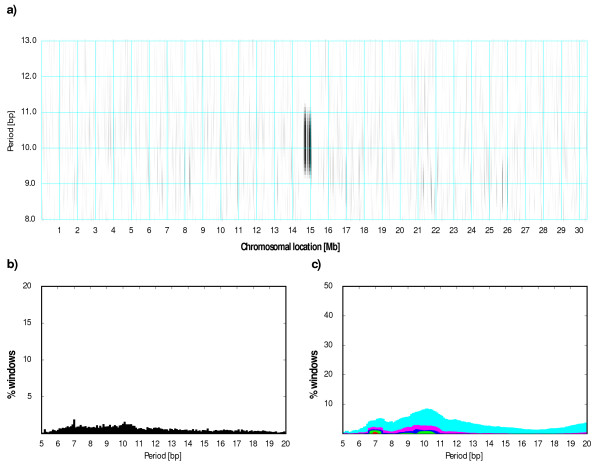
**Periodicity scan of the *A. thaliana *chromosome 1**. See legend to Figure 2.

The periodicity scan of the *A. thaliana *chromosome 1 shows a strong periodic signal with ~10 bp period restricted to the centromeric region (Figure 6). The postprocessing identifies mostly pseudogenes and a concentration of gypsy-like retrotransposons within this periodic segment (data not shown). However, the periodicity is not directly linked to gypsy elements because many gypsy-like transposons are also located outside this periodic region. The overlap of this periodic segment with the centromere suggests that the *A. thaliana *chromosome 1 centromere contains a large amount of intrinsically bent DNA. We could not verify if other *A. thaliana *chromosomes also have centromeres with a strong sequence periodicity because the centromeres in the other chromosomes were not sequenced.

## Conclusion

Intrachromosomal heterogeneity of DNA curvature-related sequence periodicity can be indicative of functional differences among distinct sections of chromosomes, differences in gene expression patterns, differences in chromatin modifications, and possibly differences in nucleoid structure or predominant mode of supercoiling [4-7,11,12,15,22]. Significant variations in the sequence periodicity also exist among different genomes, which could relate to differences in the DNA organization in the cell and possibly reflect specific environmental adaptations [4,7,9]. The PerPlot and PerScan tools presented here are suitable for analysis of interchromosomal differences as well as intrachromosomal heterogeneity of DNA curvature-related periodic patterns. These tools provide a quick and convenient way to detect anomalous regions of chromosomes that could have unusual structural and functional properties and/or distinct evolutionary history.

## Availability and Requirements

We expect that most users will access the software via web interfaces at http://www.cmbl.uga.edu/software.html. The maximum length of the analyzed sequence for the online version is limited to 50 Mb, which is sufficient for all prokaryotic and most eukaryotic chromosomes. For analysis of longer sequences, the users can download the program source codes at http://www.cmbl.uga.edu/downloads/programs/SequencePeriodicity/ and modify the maximum sequence length. The programs are written in C and distributed under the terms of the GNU General Public License. The programs were developed and tested on Red Hat Enterprise Linux operating system.

## Competing interests

The authors declare that they have no competing interests.

## Authors' contributions

JM conceived the project, designed software, analyzed data, and drafted the manuscript. TC and AB designed software and contributed to the preparation of the manuscript. All authors read and approved the final manuscript.
